# The Role of Religious Comfort and Strain on Social Well-Being among Emerging Adults in Poland: Serial Mediation by Meaning Making Processes

**DOI:** 10.1007/s10943-024-02102-8

**Published:** 2024-08-20

**Authors:** Dariusz Krok, Małgorzata Szcześniak, Beata Zarzycka

**Affiliations:** 1 Institute of Psychology, Opole University, Plac Staszica 1a, 45-052 Opole, Poland; 2 Institute of Psychology, University of Szczecin, Krakowska 69, 71-017 Szczecin, Poland; 3 Institute of Psychology, The John Paul II Catholic University of Lublin, Al. Raclawickie 14, 20-950 Lublin, Poland

**Keywords:** Religious comfort, Religious strain, Meaning-making, Meaning in life, Social well-being

## Abstract

Religious struggles tend noticeably to influence the sphere of social well-being in emerging adulthood as individuals modify their religious beliefs and practices, form personal identity patterns, and rediscover new life purpose and values. The aim of the current study was to investigate whether meaning-making and meaning in life (presence and search) can serially mediate the hypothesized links between religious comfort vs. strain and social well-being. Self-report measures of religious struggles, meaning-making, meaning in life, and social well-being were completed by 368 emerging adults (aged 18 to 29, 52.2% female). The serial mediation analysis showed that the relationship between religious comfort vs. strain and social well-being was mediated by meaning-making and presence of meaning, but not by a search for meaning. These results suggest that religious struggles may represent unique aspects of developmental spiritual processes in emerging adulthood with implications for social well-being.

## Introduction

### Religiosity and Well-Being in Emerging Adulthood

Emerging adulthood as a distinctly new developmental stage (18–29 years) introduced by Arnett (2000, 2004) has been receiving increasing attention in the field of psychological research on religion and development (Haney & Rollock, 2020; Upenieks, 2021). This is due, in part, to the fact that emerging adulthood is a period distinctly marked by dynamic changes in religious convictions and behavior which originate from a sense of instability, religious identity exploration, changes in preferred value systems, and a reinterpretation of worldviews.

Recent research has shown that religious and spiritual development tends to peak in emerging adulthood as individuals begin to alter their approach to religious practices, reflect upon meaning in life, and reformulate religious belief systems (Hwang et al., 2022; Stearns & McKinney, 2021). Although previous findings have demonstrated clear links between religiosity and well-being in emerging adults (Cook et al., 2014; Kirk & Lewis, 2013), little is known about associations between religious comfort vs. strain and social well-being and their underlying psychological mechanisms, especially in the domain of meaning-making.

In light of current research, it seems obvious that religion can play both a positive and negative role during emerging adulthood as religious beliefs and behaviors are closely interconnected with mental and physical health (Hart & Koenig, 2020). Exline (2002), Exline & Rose (2013) conceptualized adaptive and maladaptive functions of religion in terms of religious comfort and strain. Religious comfort reflects feelings of being loved by God, an inner experience of belonging to a religious community, and a sense of being forgiven, whereas religious strain (struggle) represents disappointment, anger and mistrust toward God, feelings of alienation from one’s religious community, and pervasive feelings of fear and guilt. Religious comfort can be especially important for emerging adults because of providing a sense of identity, meaning and coping at this stage of development (Bassett & Bussard, 2021). Religion can provide a sense of identity and belonging, thus helping emerging adults to identity exploration and development in the face of uncertainty (Exline & Rose, 2013; Molteni et al., 2021).

### Direct Associations between Religious Comfort and Strain and Well-Being

Research in university students demonstrated that religious comfort was related to positive outcomes in the form of better physical health and higher perceived posttraumatic growth (Cook et al., 2013). Religious comfort was also positively associated with self-esteem in a group of Polish young adults aged 18 to 25 (Szcześniak et al., 2022). In a clinical sample of women with cancer, religious comfort was negatively related to anxiety and positively related to hope (Zarzycka et al., 2019).

A different configuration of findings has emerged from research focusing on religious strain. The results of a longitudinal study conducted among US undergraduates indicated that religious struggles were positively associated with depressive symptoms and anxiety symptoms; they were also negatively related to spiritual adjustment and benevolent God representations (Sanders, 2016). In another sample of US undergraduates (mean age of 19, *SD* = 2.3), both current and lifetime religious struggles were negatively related to the three positive indicators of well-being: hedonic, social, and psychological (Wilt et al., 2017).

Similar patterns have been found in other countries. A higher level of religious struggle (i.e., strain) was negatively associated with satisfaction with life in a group of Muslim international students; however, this association was moderated by nationality: the correlation was significant among Palestinians and Turks, but was non-significant among Malaysians (Abu-Raiya et al., 2018). A recent meta-analysis of longitudinal studies on religious struggles (strains) indicated its impact on psychological adjustment (Bockrath et al., 2022). Specifically, the results showed that religious/spiritual struggles significantly predicted increases in negative psychological adjustment, but did not predict any significant changes in positive psychological adjustment. This may suggest that religious strain leads to worsening psychological adjustment, but it does not decrease positive adjustment effects.

However, some studies present a more nuanced picture of religious strain as not always leading to negative outcomes in well-being. Examining God’s role in suffering among college undergraduates, Wilt et al. (2016) found that individuals who perceived suffering as part of God’s benevolent project had a higher level of well-being, whereas those who considered a non-benevolent God causing stress and suffering were characterized by lower levels of well-being and greater religious struggle. A surprising observation, however, was that undergraduates who believed that suffering was part of God’s benevolent plan also had higher religious struggle. This could be explained by the interpretation that experiencing anger toward God, which is part of religious strain, is an important component of growing and maturing faith. Indeed, religious strain can be a beneficial factor for spiritual growth and development (Büssing et al., 2013), particularly when people are able to locate and discover positive elements emerging from difficult and challenging experiences.

### The Indirect Relationship of Religious Comfort and Strain with Well-Being

There is empirical evidence suggesting that the relationship of religious comfort and strain with well-being indicators is mediated by other psychological factors. Self-esteem has been found to mediate the relationships between religious comfort and life satisfaction and between religious strain (i.e., fear/guilt and life satisfaction, negative emotions toward God, and negative social interactions surrounding religion) and life satisfaction among adults (Szcześniak & Timoszyk-Tomczak, 2020).

Examining associations between religious struggles and psychological well-being among adults, Zarzycka and Puchalska-Wasyl (2020) demonstrated that internal dialogues were mediators in these associations. More specifically, three types of internal dialogue—ruminative, supportive, and social simulation dialogues—mediated the relationship between religious struggles and psychological well-being. However, their mediational effects were different: while religious struggle reduced psychological well-being through its effect on ruminative dialogues, it enhanced well-being through its effect on supportive and social dialogues. In young adults, the relationships between religious comfort and self-esteem and between negative emotions toward God (a component of religious strain) and self-esteem were mediated by dispositional gratitude (Szcześniak et al., 2022).

Mediating relationships between religious comfort vs. strain and well-being indicators also occurred in clinical groups. In cancer patients, social support, optimism, and self-efficacy were mediators in the associations between religious struggle and psychosocial adjustment (Pearce et al., 2006). Hope was also found to mediate the relationship between religious comfort and anxiety in women with cancer: patients with higher religious comfort experienced stronger hope, which in turn was related to lower anxiety (Zarzycka et al., 2019). This more nuanced understanding of religious comfort and strain laid emphasis on the importance of explanatory models that might account for the multifaceted role played by religion in coping with uncertainty and distress.

### The Role of Meaning-Making in Religious Struggles and Well-Being

Recent studies have suggested that meaning-making can be a prospective factor in explaining the mediating nature of the relationship between religious struggles and well-being (Appel et al., 2020; Stauner et al., 2020). A potential rationale may be based on the observation that stressful and challenging life events often undermine religious aspects of meaning systems (Park, 2020; Park et al., 2017), which can be reflected in the sphere of religious comfort and strain. In such situations, individuals often seek spiritual support that can counterbalance emerging tension and distress. In addition, religiousness can be seen as a meaning system that provides guidance in managing challenging and demanding life events (Park, 2013; Park & Van Tongeren, 2023). When dealing with problematic situations, individuals are likely to rely on religious meaning systems to develop a coherent and satisfying understanding of their lives. Through the process of making-meaning, individuals can better understand both themselves and their religious beliefs and values, which contributes to well-being.

This assumption has received empirical backing in studies examining meaning-making as a mediator. In an adult group, most of whom were in the period of emerging adulthood (M = 24 years), meaning-making was a mediator between only one dimension of religious struggle, i.e., doubt, and life satisfaction (Zarzycka & Zietek, 2019). Individuals with greater religious doubt were characterized by stronger meaning-making activity, which in turn was associated with their higher life satisfaction. Meaning-making also mediated the relationship between religious struggles and psychological well-being in a group of adults (Zarzycka et al., 2020). Greater religious struggle was related to greater meaning-making, which, in turn, was related to higher levels of psychological well-being. These studies, however, only took into account the mediating role of meaning-making and did not measure a level of meaning in life (presence of meaning in life), which, according to the meaning-making model, is the outcome of meaning-making processes.

### The Explanatory Function of the Meaning-Making Model

The meaning-making model, which has been successfully adopted in research on religion and mental health (Davis et al., 2019; Krok et al., 2021; Park, 2020), posits that individuals respond to stressful events based on the discrepancy between their global beliefs and goals and the assessed situational meaning of the event (Park, 2010, 2017). This discrepancy, in turn, activates a process of meaning-making, which refers to efforts to restore coherence between the pre-existing global meaning and the assessed meaning attributed to the event. Meaning-making, being adaptive in nature, can result in many different outcomes—changes in one’s global meaning (Park, 2016), alterations in one’s appraisal of the situation (Taves et al., 2018), or increased well-being (Krok & Telka, 2019). These changes can also incorporate an increase in presence of meaning in life as people can derive meaning from their coping activities and life experiences, including religious struggle. Based on the meaning-making model, it is thus possible to assume a serial mediation in which meaning-making will precede any changes in meaning of life (both presence and search).

Given the close documented associations between religion and meaning in life (Krause et al., 2019; Park & Van Tongeren, 2023), a constructive meaning-making process can lead to an increase in presence of meaning in life, which, in turn, will be related to better psychosocial adjustment. Examining changes in religious doubt and mental health in emerging adulthood, Upenieks (2021) showed that meaning in life (presence of meaning) mediated the relationship between increases, but not decreases, in religious doubt and mental health. The presence of meaning in life has also consistently been found to mediate relationships between religiosity and different indicators of well-being (Steger & Frazier, 2005; You & Lim, 2019).

### The Present Study

The present study aims to examine relationships between religious comfort vs. religious strain and social well-being within the mediational role of meaning-making processes, with meaning-making and meaning in life serving as multiple serial mediators. Based on previous studies (Krause et al., 2019; Upenieks, 2021; Zarzycka & Zietek, 2019) and the meaning-making model (Park, 2010, 2017), it was hypothesized that religious comfort is positively related to social well-being and this relationship is serially mediated by meaning-making and meaning in life. It was also hypothesized that religious strain is negatively associated with social well-being and this relationship is serially mediated by meaning-making and meaning in life. In addition, religious comfort and strain are expected to have different mediating effects.

## Materials and Method

### Participants

Initially, a total of 429 participants aged 18 to 29 (representing a period of emerging adulthood) were recruited: 46 declined to participate and 15 did not complete the questionnaires in full. The final sample thus consisted of 368 participants, yielding a final participation ratio of 85.8 percent. The group included 192 women (52.2%) and 176 men (47.8%), with a mean age of *M* = 23.18 (*SD* = 3.77). There were no statistically significant differences between women and men in terms of age. Approximately half of the participants attended a university/high school, and half had a full-time job. Most of those surveyed (71.5%) were single, while the remaining 28.5% of participants had a spouse or partner. In terms of education, the most numerous group were high school graduates (general or professionally profiled). Most participants (82.6%) were Roman Catholics; 7.8 percent were Protestants, and 9.6 percent described themselves as agnostics or atheists. In general, this sample reflects Poland’s sociodemographic characteristics regarding religion, education, marital and employment status among emerging adults (DSP, 2022). The exact detailed sociodemographic data are presented in Table 1.

**Table 1:** Participant sociodemographic characteristics (*N* = 368)

Characteristics	*M*	*SD*
Age	23.18	3.77
	*N*	*%*
Sex		
Male	176	47.8
Female	192	52.2
Employment status		
Unemployed	121	32.9
Full time work	247	67.1
Marital status		
Married/Living with partner	105	28.5
Single	263	71.5
Educational attainment		
Basic vocational education	69	18.7
High school education	224	60.9
University education	75	20.4
Child		
Yes	71	19.2
No	297	80.8
Religious denomination		
Roman Catholics	304	82.6
Protestants	29	7.8
Agnostics or atheists	35	9.6

### Procedure

Inclusion in the study was restricted to individuals representing the period of emerging adulthood (i.e., from 18 to 29 years). The study was conducted during classes in colleges/universities, or at work/home in southern parts of Poland. Respondents were recruited through the Internet or personal contact; the snowball method was applied. In the first case, the researchers sent an invitation letter to participate in the study to their friends via email; these individuals then forwarded the invitation to their friends. In the second case, the researchers personally contacted potential participants who expressed interest in taking part in the study. When respondents consented to the study, research assistants provided them with a set of printed or online questionnaires. Participation in the study was voluntary and anonymous; informed consent was also obtained from the subjects. The aim was to cover a wide range of sociodemographic characteristics for a representative sample of emerging adults. Participants were able to forgo the study at any stage and potential queries regarding the study were answered by research assistants. The research received a positive evaluation from the Ethical Institutional Board at the first author’s university.

### Measures

#### Religious Comfort and Strain

The Religious Comfort and Strain Scale (RCSS) was used to assess the level of religious comfort and strain (Exline et al., 2000). The scale comprises 24 items, with a one-dimensional religious comfort subscale (10 items) and three subscales representing religious strain (14 items): (1) negative emotions toward God, (2) fear/guilt, and (3) negative social interactions surrounding religion. Each item is rated on a ten-point scale from 1 (not at all) to 10 (extremely), higher scores indicating higher levels of religious comfort or strain. Cronbach's coefficients for these variables and the subsequent ones of the current study are shown in Table 2.

**Table 2:** Mean scores, standard deviations, and zero-order Pearson correlation coefficients between study variables (*N* = 368)

Variables	*Cronbach's alfa*	*M*	*SD*	1	2	3	4	5
1. Religious comfort	.78	6.69	1.96	–				
2. Religious strain	.81	5.75	2.11	−.46***	–			
3. Meaning making	.85	4.06	1.31	.28***	−.36***	–		
4. Presence of meaning	.81	4.88	.96	.24***	−.34***	.48***	–	
5. Search for meaning	.83	4.34	.93	.02	.44***	−.17**	−.25***	–
6. Social well-being	.87	4.62	.70	.16**	−.14*	.44***	.46***	−.01

^*^ *p* < .05; ** *p* < .01; *** *p* < .001

#### Social Well-being

The Social Well-Being Scale (SWB; Keyes, 1998) was used to evaluate the level of social well-being, conceptualized as the assessment of one’s situation and functioning in society, with social challenges as criteria that people use to evaluate their life quality. The scale includes 35 items forming five dimensions: social integration, social acceptance, social contribution, social actualization, and social coherence. Their sum gives a total score, which was employed in this study. The responses are on a five-point scale ranging from 1 (strongly disagree) to 5 (strongly agree). Higher scores indicate higher levels of social well-being. The Cronbach's coefficient for the current study was 0.87 (total score).

#### Meaning-making

The Meaning Making Questionnaire (MMQ; Krok & Zarzycka, 2020) was applied to assess meaning-making, which can be defined as one’s cognitive capacity to comprehend and assimilate challenging or ambiguous life events into coherent structures of personal meaning, beliefs, and goals. The questionnaire has been used in previous studies that examined the mediational associations between risk perception of Covid-19, religiosity, and subjective well-being in emerging adults (Krok et al., 2023), and between social support and illness acceptance among cancer patients (Krok et al., 2024). Their results showed that it was a reliable and valid measure. The questionnaire consists of eight items which are assessed on a five-point Likert scale from 1 (never) to 5 (very often). A higher score signifies a higher intensity of meaning-making in which people engage in response to life demands. The Cronbach’s coefficient for the present study was 0.85.

#### Meaning in Life

The Meaning in Life Questionnaire (MLQ; Steger et al., 2006) assesses meaning in life, understood as the extent to which people comprehend and make sense of their lives. The questionnaire enables measurement of meaning in life within two dimensions: the presence of meaning, which evaluates the extent to which participants perceive their lives as meaningful; and the search for meaning, which gauges the extent to which individuals are actively seeking meaning or purpose in their lives. Each dimension is evaluated by five items rated on a seven-point Likert scale ranging from 1 (absolutely untrue) to 7 (absolutely true). The Cronbach’s coefficients in the current study were 0.81 for the presence subscale and 0.83 for the search scale.

### Statistical Analyses

Based on the recommendations of Koopman et al. (2015) and Preacher et al. (2007) regarding sample size in mediation model analyses, G*Power software was used to calculate a priori the minimum sample size required for the model proposed in our study. The following criteria were adopted: power level (1 – β), prespecified significance level (α = 0.05), and test power ((1 – β) = 0.90) (Faul et al., 2009). The following type of analysis for a priori power analysis G* was applied: F test; linear multiple regression: fixed model, R2 deviation from zero; type of power analysis: A priori. The results showed that the sample n > 344 would be sufficient to exert significant effects. Next, as all variables were measured using self-report questionnaires, we wanted to exclude the possibility that our study is biased by common method variance (Podsakoff et al., 2003). The Harman's one-factor test determined that a single factor explained 14.25 percent of the variance and that all items formed 21 separate dimensions, which also indicates that the present data were not affected by common method error.

The statistical analysis was conducted in two stages. Firstly, descriptive statistics and Pearson’s *r* correlations were computed among the key study variables (religious comfort, religious strain, meaning-making, presence of meaning, search for meaning, and social well-being) using IBM SPSS Statistics 27. Secondly, PROCESS macro v4.0 (Hayes, 2018, model 81) was used to perform multiple serial mediation analyses; the bootstrapping procedure was based on the following parameters: samples = 10,000; 95% bias-corrected confidence intervals.

## Results

### Preliminary Analyses

First, means, standard deviations, and Pearson correlation coefficients were calculated (Table 2). The results indicated that religious comfort was negatively related to religious strain but positively related to meaning-making, presence of meaning, and social well-being. In contrast, religious strain was negatively correlated with meaning-making, presence of meaning, and social well-being but positively correlated with search for meaning. Meaning-making was positively related to presence of meaning and social well-being, and negatively related to search for meaning. Presence of meaning, but not search for meaning, was positively correlated with social well-being.

## Mediation Analysis

Mediation analysis with parallel and serial mediators (Model 81) and the bootstrapping procedure was applied to calculate mediational effects in two different models (Hayes, 2018). They included three categories of variable: (1) religious comfort and religious strain as independent variables; (2) meaning-making, presence of meaning, and search for meaning as mediators; and (3) social well-being as the dependent variable.

**Fig. 1:**
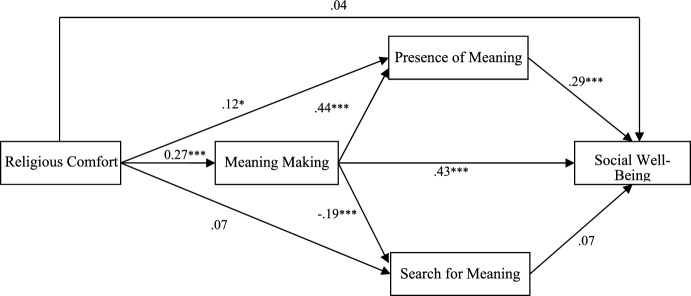
Mediation effects of meaning making and meaning in life on the relationship between religious comfort and social well-being (Standardized Regression Coefficients)

In Model 1 (Fig. 1), with religious comfort as the independent variable, four of the five potential mediating effects in the religious comfort–social well-being relationship were significant (Table 3).

**Table 3:** Direct and indirect effects for religious comfort and strain (standardized coefficients)

Model pathways	Effect	SE	LLCI	ULCI
**Model 1:** Religious comfort as independent variable				
Direct effect				
Religious comfort → Social well-being	.03	.02	−.04	.02
Indirect effects				
Religious comfort → Meaning making → Social well-being	.12	.03	.08	.17
Religious comfort → Presence of meaning → Social well-being	.03	.02	.01	.07
Religious comfort → Search for meaning → Social well-being	.01	.01	−.01	.03
Religious comfort → Meaning making → Presence of meaning → Social well-being	.04	.01	.01	.06
Religious comfort → Meaning making → Search for meaning → Social well-being	−.01	.004	.02	−.002
Total indirect effect	.19	.03	.13	.25
Specific indirect effect contrast				
Presence of meaning *and* Search for meaning	.03	.02	−.01	.07
Meaning making and Presence of meaning *and* Meaning making and Search for meaning	.04	.01	.02	.07
**Model 2:** Religious strain as independent variable				
Direct effects				
Religious strain → Social well-being	.06	.01	−.01	.04
Indirect effects				
Religious strain → Meaning making → Social well-being	−.16	.03	−.22	−.11
Religious strain → Presence of meaning → Social well-being	−.06	.02	−.01	−.02
Religious strain → Search for meaning → Social well-being	.06	.03	.01	.11
Religious strain → Meaning making → Presence of meaning → Social well-being	−.04	.01	−.07	−.02
Religious strain → Meaning making → Search for meaning → Social well-being	−.001	.002	−.01	.003
Total indirect effect	−.20	.05	−.29	−.12
Specific indirect effect contrast				
Presence of meaning *and* Search for meaning	−.11	.03	−.18	−.05
Meaning making and Presence of meaning *and* Meaning making and Search for meaning	−.04	.01	−.07	−.02

Firstly, meaning-making and presence of meaning were parallel mediators in the religious comfort–social well-being relationship. More specifically, religious comfort was positively related to meaning-making (*B* = 0.27, t = 5.55, *p* < 0.001), which in turn was positively related to social well-being (*B* = 0.43, t = 8.98, *p* < 0.001). Religious comfort was also positively related to presence of meaning (*B* = 0.27, *t* = 5.55, *p* < 0.001), which in turn was positively related to social well-being (*B* = 0.29, *t* = 5.89, *p* < 0.001).

In contrast, the indirect effect of search for meaning in the religious comfort–social well-being relationship was not significant. Meaning-making and presence of meaning were serial mediators in the relationship of religious comfort with social well-being. Specifically, meaning-making was positively related to presence of meaning (*B* = 0.44, *t* = 9.46, *p* < 0.001), which in turn was positively related to social well-being (*B* = 0.29, *t* = 5.89, *p* < 0.001). The mediating effect of meaning-making and search for meaning in the religious comfort–social well-being relationship turned out to be non-significant. Furthermore, the total indirect effect of parallel and serial mediation by meaning-making, presence of meaning, and search for meaning was significant.

To compare the separate mediation effects exerted by presence of meaning and search for meaning, indirect effect contrast procedures were used, the results of which are presented in Table 3. For parallel mediation, there was no significant difference in the mediating powers of presence of meaning and search for meaning in the relationship of religious comfort with social well-being. However, for serial mediation, the indirect effect contrast test showed significant differences in the mediating powers of meaning-making and presence of meaning vs. meaning-making and search for meaning. In Model 1, the direct effect of religious comfort on social well-being was not significant (Fig. 1).

**Fig. 2:**
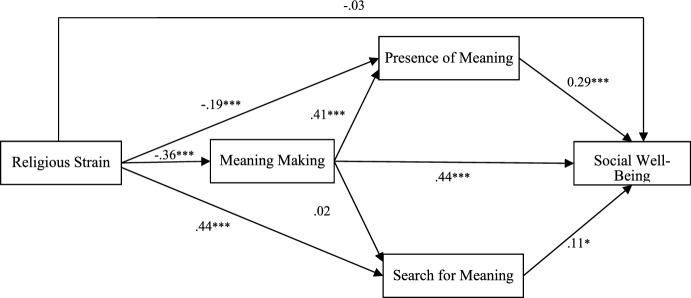
Mediation effects of meaning making and meaning in life on the relationship between religious strain and social well-being (Standardized Regression Coefficients)

In Model 2 (Fig. 2), with religious strain as the independent variable, four of the five potential mediating effects in the religious strain–social well-being relationship were significant (Table 3). Firstly, meaning-making and presence of meaning were parallel mediators in the relationship of religious strain with social well-being. However, their mediating effects were significantly different. Religious strain was negatively related to meaning-making (*B* = -0.36, t = -7.41, *p* < 0.001), which in turn was positively related to social well-being (*B* = 0.44, t = 9.01, *p* < 0.001). Similarly, religious strain was negatively related to presence of meaning (*B* = -0.19, *t* = -3.98, *p* < 0.001), which in turn was positively related to social well-being (*B* = 0.29, *t* = 6.01, *p* < 0.001).

In contrast, religious strain was positively related to search for meaning (*B* = 0.44, *t* = -3.98, *p* < 0.001), which in turn was positively related to social well-being (*B* = 0.11, *t* = 2.14, *p* < 0.05). Meaning-making and presence of meaning were serial mediators in the relationship of religious strain with social well-being: meaning-making was positively related to presence of meaning (*B* = 0.41, *t* = 8.55, *p* < 0.001), which in turn was positively related to social well-being (*B* = 0.29, *t* = 6.01, *p* < 0.001). The serial mediating effect of meaning-making and search for meaning turned out to be nonsignificant. Finally, the total indirect effect of parallel and serial mediation by meaning-making, presence of meaning, and search for meaning was significant (Fig. 2).

As our model included both parallel and serial mediators, an effect contrast method was used to estimate differences between the separate mediation effects (Hayes, 2018). The results of indirect effect contrast demonstrated that for parallel mediation, the mediating powers of presence of meaning and search for meaning in the relationship of religious strain with social well-being were significantly different. For serial mediation, the contrast test also indicated significant differences in the mediating powers of meaning-making and presence of meaning vs. meaning-making and search for meaning (Table 3). The direct effect of religious comfort on social well-being was not significant in Model 2.

Finally, as search for meaning played no role in the above model with parallel and serial mediators, we decided to rerun the analyses without search for meaning (Model 6, Hayes, 2018). However, compared to the original model, which included search for meaning, there were no significant differences in mediating effects between the two models.

## Discussion

The present study aimed to investigate the relationship between religious comfort vs. strain and social well-being in emerging adulthood within a parallel-serial model in which meaning-making and meaning in life were mediators. This enabled us to examine the mediating functions of meaning-making and meaning in life as the underlying psychological mechanisms connecting religious comfort and strain to emerging adults’ well-being within the meaning-making model. To our knowledge, this is the first study to demonstrate indirect associations between religious comfort vs. strain and social well-being, which occurred through meaning-making processes in an emerging adult sample.

### Religious Comfort vs. Strain and Social Well-Being

The results that religious comfort was positively related to social well-being whereas religious strain was negatively related are consistent with our hypothesis and with previous findings connecting religious comfort to better physical and mental health (Cook et al., 2013; Szcześniak et al., 2022; Zarzycka et al., 2019) and religious strain to negative well-psychosocial adjustment (Bockrath et al., 2022; Sanders, 2016). This justifies examining them as two different but interrelated constructs in the sphere of religious struggles.

Yet, our study extends the existing literature by demonstrating that both religious comfort and strain are interwoven within the characteristic facets of emerging adulthood—identity exploration, instability, and self-focus. During this period, individuals experience many dynamic changes related to the formation of personal identity patterns, the construction of future professional and family relationships, and the development of responsibility for self and others (Arnett, 2004; Haney & Rollock, 2020). Our findings demonstrate that personal experiences of being loved by God, belonging to a religious community, and feeling inner peace can help emerging adults develop and maintain positive interactions with other people. In contrast, persistent feelings of anger towards God, alienation from a religious community, and moral guilt tend to prevent emerging adults from establishing satisfying and fulfilling social relationships. To some extent, therefore, maintaining a balance between religious comfort and strain is important for shaping optimal levels of social well-being. This is because religion can fulfill both positive and negative roles in social behavior. On one hand, it offers social support through involvement in religious communities, while on the other hand, it can generate tensions stemming from societal judgments and norms.

In line with previous research, no significant differences were found in the effect sizes of religious comfort vs. strain on social well-being (Effects = 0.04 and -0.03, respectively) (Zarzycka et al., 2019). This may suggest that during emerging adulthood, experiencing both affirmative and tense feelings toward God and the religious community is comparatively important for social well-being.

One potential explanation can be found in specific developmental features of emerging adulthood in which dynamic changes frequently occur within religiosity. Although religious activity tends to decline during this period (Upenieks, 2021), many people still seek emotional stability, overarching values, and satisfying answers to existential doubts in religious beliefs and activities. As a consequence, positive and negative feelings toward God and the religious community (i.e., religious comfort vs. strain) are likely to coexist in the minds of individuals and jointly influence social interactions. This explanation is supported by findings showing that adaptive and maladaptive functions of religion can manifest themselves in the form of religious comfort and strain (Bockrath et al., 2022; Exline & Rose, 2013). Therefore, our results further emphasize the importance of investigating the sequelae of comfort and strain jointly. At the macro level, religious comfort is beneficial and religious stress is detrimental as they have a different relationship with well-being (positive vs. negative).

### Indirect Associations of Religious Comfort vs. Strain with Social Well-Being

Consistent with our hypothesis, meaning-making and presence of meaning in life, but not search for meaning, were serial mediators in the relationship between religious comfort and social well-being; the presence and search factors were investigated within two separate models. Religious comfort was positively related to meaning-making, which, in turn, was positively related to presence of meaning and then positively to social well-being. This finding is in line with previous studies that demonstrated a significant role of meaning structures (i.e., meaning-making and meaning in life) in associations between religion and well-being (You & Lim, 2019; Zarzycka et al., 2020). During the period of emerging adulthood, it is particularly important to discover and have a satisfying level of meaning in life (presence of meaning), as it enables individuals to interpret and organize their daily experiences, form a personal identity, and pursue overarching goals and values (Martela & Steger, 2022). Furthermore, this finding suggests that religious comfort offers a potent source of meaning that strengthens young adults’ abilities to develop and maintain positive interactions with other people and communities.

Meaning-making and presence of meaning, but not search for meaning, were serial mediators in the relationship between religious strain and social well-being, which partially confirms our last hypothesis. Contrary to religious comfort, religious strain was negatively related to meaning-making, which, in turn, was positively related to presence of meaning and then positively to social well-being. This result is consistent with recent studies showing that meaning-making mediated the relationship between religious struggle and mental health in adults (Zarzycka & Zietek, 2019) and emerging adults (Upenieks, 2021). The effective process of making meaning was also beneficial for obtaining higher presence of meaning in life, but not for seeking it, which led to fulfilling and satisfying social interactions (i.e., social well-being).

The result regarding the mediating role of meaning-making differed somewhat from the earlier findings obtained by Zarzycka et al. (2020), however. In their study, despite confirming the mediating function of meaning-making in associations between religious struggles and well-being, higher levels of religious struggle were associated with higher meaning-making, whereas in our study, the relationship was the opposite—higher religious strain was related to lower meaning-making.

This difference can be explained in two ways. Firstly, in Zarzycka et al.’s (2020) study, the age range of the participants was much wider (17–78 years) than in the present study, which may have influenced the process of meaning-making, as older participants tend to experience more religious struggles (Exline & Rose, 2013). Secondly, the first study examined the serial mediation of religious support and meaning-making in the relationship between religious struggles and psychological well-being. The use of different factors in the aforementioned study (i.e., social support and psychological well-being) may have altered the association of religious struggles with meaning-making. As demonstrated by Mosley-Johnson et al. (2019), there are noticeable differences between psychological and social well-being, especially in terms of factors related to the importance of relationships with others and deriving satisfaction from societal values.

Our study extends previous research by providing empirical evidence supporting some main propositions of the meaning-making model (Park, 2010, 2016). The influence of religious comfort and strain on social well-being was explained by the extent to which meaning-making led to higher meaning in life, which, in turn, was related to more satisfying social well-being. These findings show that young adults are able to build and develop satisfying relationships with others on the basis of positive religious experiences provided that they constructively understand their current situation and create personal sets of beliefs and goals, which leads to finding meaning in life.

This interpretation is supported by research (Krok et al., 2021), demonstrating that religious beliefs can influence the ways in which individuals deal with challenging events through successful meaning-making. In addition, the subjective meaning emerging adults derive from their attempts to resolve potential ambiguities and concerns, and their efforts to search for meaning in life, seem to explain the positive relationship between religious comfort and social well-being.

The meaning-making model posits that meaning-making is adaptive when satisfactory meanings are produced (Park, 2013; Park & Van Tongeren, 2023). In our study, meaning-making was positively related to presence of meaning but negatively related to search for meaning. Furthermore, the mediating effect of meaning-making and search for meaning was non-significant. This indicates that in emerging adulthood, meaning-making is only adaptive and leads to higher well-being when it results in a higher level of meaning in life. As a consequence, general meaning in life can be capable of increasing social well-being (Martela & Steger, 2022). However, an inability to develop and obtain a coherent view of reality successfully leads to existential ambiguities and doubts, entailing higher search for meaning.

In the absence of a clear understanding of reality, individuals may question their fundamental beliefs, values, and life goals, as well as struggle to make sense of their experiences and the world around them. Consequently, this can provoke doubts and uncertainty related to meaning of life.

### Limitations and Future Directions

Several limitations of the present study should be acknowledged. Firstly, our study has a cross-sectional design, which forbids conclusions regarding directionality in the investigated associations. It is possible that religious comfort vs. strain and meaning in life (presence of meaning) interact over time, which could modify the results obtained by using the cross-sectional data. Future research with longitudinal data should examine this possibility.

Secondly, religious comfort and strain are complex and multifaceted phenomena that are most likely to depend on overall distress, anxiety, and existential uncertainty (Exline, 2002). As we did not measure this variable, the level of religious comfort vs. strain measured in our study could be a consequence not only of one’s attitude toward God and a religious community, but also of other incidental life circumstances.

Finally, only self-report instruments were used, which places our study at risk of participants providing answers under the influence of memory bias or social desirability (Taves et al., 2018). Other forms of assessing meaning in life or religious struggle (e.g., quasi-experiments or indirect measures) would be desirable to reduce the impact of social subjectivity.

### Implications for Interventions and Clinical Practice

The study also has practical, clinical implications as it provides clergy, clinicians and therapists with additional knowledge about the role of different dimensions of religiosity (i.e. comfort and strain) in functioning in society and responding to social challenges. The counseling offered to emerging adults in times of religious doubt and crisis should, above all, consider meaning structures, bot at the level of processes (i.e. meaning-making), and personal resources (especially, presence of meaning in life) (Hwang et al., 2022; Park & Van Tongeren, 2023). Furthermore, therapeutic interventions that increase a sense of God’s love and belonging to a religious community along with activities directed at discovering fulfilling goals and values would help form positive and satisfying patterns of social relationships in emerging adulthood.

## Conclusion

Despite these limitations, the current study extends our understanding of the relationships between religious comfort vs. strain and social well-being by showing that meaning-making and presence of meaning in life are serial mediators in these relationships. Religious comfort was positively related, and religious strain negatively related, to meaning-making, which, in turn, was positively related to presence of meaning and then to social well-being. In contrast, no mediational effects were found for search for meaning. These findings can be fully understood within the meaning-making model that offers a valuable theoretical framework in the field of religion and well-being. Taken together, the findings provide a broader vision of religion and well-being by revealing psychological mechanisms that indicate meaning structures as underlying factors responsible for both the benefits and costs of religious behavior.

## Data Availability

The data presented in this study are available in OSF HOME at: https://osf.io/5acz4/

## References

[CR1] Abu-Raiya, H., Ayten, A., Agbaria, Q., & Tekke, M. (2018). Relationships between religious struggles and well-being among a multinational Muslim sample: A comparative analysis. *Social Work,**63*(4), 347–356. 10.1093/sw/swy031 30085296 10.1093/sw/swy031

[CR2] Appel, J. E., Park, C. L., Wortmann, J. H., & van Schie, H. T. (2020). Meaning violations, religious/spiritual struggles, and meaning in life in the face of stressful life events. *The International Journal for the Psychology of Religion,**30*(1), 1–17. 10.1080/10508619.2019.1611127

[CR3] Arnett, J. J. (2000). Emerging adulthood: A theory of development from the late teens through the twenties. *American Psychologist,**55*(5), 469–480. 10.1037/0003-066X.55.5.469 10842426

[CR4] Arnett, J. J. (2004). *Emerging adulthood: The winding road from the late teens through the twenties*. Oxford: Oxford University Press.

[CR5] Bassett, J. F., & Bussard, M. L. (2021). Examining the complex relation among religion, morality, and death anxiety: Religion can be a source of comfort and concern regarding fears of death. *OMEGA-Journal of Death and Dying,**82*(3), 467–487. 10.1177/0030222818819343 10.1177/0030222818819343 30572785

[CR6] Bockrath, M. F., Pargament, K. I., Wong, S., Harriott, V. A., Pomerleau, J. M., Homolka, S. J., Chaudhary, Z. B., & Exline, J. J. (2022). Religious and spiritual struggles and their links to psychological adjustment: A meta-analysis of longitudinal studies. *Psychology of Religion and Spirituality,**14*(3), 283–299. 10.1037/rel0000400

[CR7] Büssing, A., Günther, A., Baumann, K., Frick, E., & Jacobs, C. (2013). Spiritual dryness as a measure of a specific spiritual crisis in Catholic priests: Associations with symptoms of burnout and distress. *Evidence-Based Complementary and Alternative Medicine*. Article ID 246797. 10.1155/2013/24679 7 10.1155/2013/246797 PMC3703410 23843867

[CR8] Cook, K. V., Kimball, C. N., Leonard, K. C., & Boyatzis, C. J. (2014). The complexity of quest in emerging adults’ religiosity, well-being, and identity. *Journal for the Scientific Study of Religion,**53*(1), 73–89. 10.1111/jssr.12086

[CR9] Cook, S. W., Aten, J. D., Moore, M., Hook, J. N., & Davis, D. E. (2013). Resource loss, religiousness, health, and posttraumatic growth following Hurricane Katrina. *Mental Health, Religion & Culture,**16*(4), 352–366. 10.1080/13674676.2012.667395

[CR10] Davis, E. B., Kimball, C. N., Aten, J. D., Andrews, B., Van Tongeren, D. R., Hook, J. N., Davis, D. E., Granqvist, P., & Park, C. L. (2019). Religious meaning making and attachment in a disaster context: A longitudinal qualitative study of flood survivors. *The Journal of Positive Psychology,**14*(5), 659–671. 10.1080/17439760.2018.1519592

[CR11] DSP (2022). *Demographic situation in Poland up to 2022*. https://stat.gov.pl/obszary-tematyczne/ludnosc/ludnosc/sytuacja-demograficzna-polski-do-roku-2022,40,3.html (30.07.2024).

[CR12] Exline, J. J. (2002). Stumbling blocks on the religious road: Fractured relationships, nagging vices, and the inner struggle to believe. *Psychological Inquiry,**13*, 182–189. 10.1207/S15327965PLI1303_03

[CR13] Exline, J. J., & Rose, E. (2013). Religious and spiritual struggles. In R. F. Paloutzian & C. L. Park (Eds.), *Handbook of the psychology of religion and spirituality* (pp. 380–398). Guilford.

[CR14] Exline, J. J., Yali, A. M., & Sanderson, W. C. (2000). Guilt, discord, and alienation: The role of religious strain in depression and suicidality. *Journal of Clinical Psychology,**56*, 1481–1496. 10.1002/1097-4679(200012)56:12%3c1481::AID-1%3e3.0.CO;2-A 11132565 10.1002/1097-4679(200012)56:12<1481::AID-1>3.0.CO;2-A

[CR15] Faul, F., Erdfelder, E., Buchner, A., & Lang, A.-G. (2009). Statistical power analyses using G*Power 3.1: Tests for correlation and regression analyses. *Behavior Research Methods,**41*, 1149–1160. 10.3758/BRM.41.4.1149 19897823 10.3758/BRM.41.4.1149

[CR16] Haney, A. M., & Rollock, D. (2020). A matter of faith: The role of religion, doubt, and personality in emerging adult mental health. *Psychology of Religion and Spirituality,**12*(2), 247–253. 10.1037/rel0000231

[CR17] Hart, C. W., & Koenig, H. G. (2020). Religion and health during the COVID-19 pandemic. *Journal of Religion and Health,**59*(3), 1141–1143. 10.1007/s10943-020-01042-3 32415424 10.1007/s10943-020-01042-3 PMC7226717

[CR18] Hayes, A. F. (2018). *Introduction to mediation, moderation, and conditional process analysis: A regression-based approach* (2nd ed.). New York: The Guilford Press.

[CR19] Hwang, W., Zhang, X., Brown, M. T., Vasilenko, S. A., & Silverstein, M. (2022). Religious transitions among baby boomers from young adulthood to later life: Associations with psychological well-being over 45 years. *The International Journal of Aging and Human Development,**94*(1), 23–40. 10.1177/00914150211029892 34672211 10.1177/00914150211029892 PMC10903278

[CR20] Keyes, C. L. M. (1998). Social well-being. *Social Psychology Quarterly,**61*, 121–140. 10.2307/2787065

[CR21] Kirk, C. M., & Lewis, R. K. (2013). The impact of religious behaviours on the health and well-being of emerging adults. *Mental Health, Religion & Culture,**16*(10), 1030–1043. 10.1080/13674676.2012.730037

[CR22] Koopman, J., Howe, M., Hollenbeck, J. R., & Sin, H.-P. (2015). Small sample mediation testing: Misplaced confidence in bootstrapped confidence intervals. *Journal of Applied Psychology,**100*(1), 194–202. 10.1037/a0036635 24731180 10.1037/a0036635

[CR23] Krause, N., Hill, P. C., & Ironson, G. (2019). Evaluating the relationships among religion, social virtues, and meaning in life. *Archive for the Psychology of Religion,**41*(1), 53–70. 10.1177/008467241983979

[CR24] Krok, D., & Telka, E. (2019). The role of meaning in gastric cancer patients: Relationships among meaning structures, coping, and psychological well-being. *Anxiety, Stress, & Coping,**32*(5), 522–533. 10.1080/10615806.2019.1633578 31234657 10.1080/10615806.2019.1633578

[CR25] Krok, D., Telka, E., & Kocur, D. (2024). Perceived and received social support and illness acceptance among breast cancer patients: The serial mediation of meaning-making and fear of recurrence. *Annals of Behavioral Medicine,**58*(3), 147–155. 10.1093/abm/kaad067 38134347 10.1093/abm/kaad067 PMC10858304

[CR26] Krok, D., & Zarzycka, B. (2020). Self-efficacy and psychological well-being in cardiac patients: Moderated mediation by affect and meaning-making. *The Journal of Psychology,**154*(6), 411–425. 10.1080/00223980.2020.1772702 32484755 10.1080/00223980.2020.1772702

[CR27] Krok, D., Zarzycka, B., & Telka, E. (2021). The interplay of religious and nonreligious meaning-making on psychological well-being in gastrointestinal cancer patients. *The International Journal for the Psychology of Religion,**31*(4), 276–287. 10.1080/10508619.2020.1834746

[CR28] Krok, D., Zarzycka, B., & Telka, E. (2023). Risk perception of Covid-19, religiosity, and subjective well-being in emerging adults: The mediating role of meaning-making and perceived stress. *Journal of Psychology and Theology,**51*(1), 3–18. 10.1177/00916471221102550 37038469 10.1177/00916471221102550 PMC10076236

[CR29] Martela, F., & Steger, M. F. (2022). The role of significance relative to the other dimensions of meaning in life–an examination utilizing the three dimensional meaning in life scale (3DM). *The Journal of Positive Psychology*. 10.1080/17439760.2022.2070528

[CR30] Molteni, F., Ladini, R., Biolcati, F., Chiesi, A. M., Dotti Sani, G. M., Guglielmi, S., Maraffi, M., Pedrazzani, A., Segatti, P., & Vezzoni, C. (2021). Searching for comfort in religion: insecurity and religious behaviour during the COVID-19 pandemic in Italy. *European Societies,**23*(sup1), S704–S720. 10.1080/14616696.2020.1836383

[CR31] Mosley-Johnson, E., Garacci, E., Wagner, N., Mendez, C., Williams, J. S., & Egede, L. E. (2019). Assessing the relationship between adverse childhood experiences and life satisfaction, psychological well-being, and social well-being: United States Longitudinal Cohort 1995–2014. *Quality of Life Research,**28*(4), 907–914. 10.1007/s11136-018-2054-6 30467779 10.1007/s11136-018-2054-6 PMC6459973

[CR32] Park, C. L. (2010). Making sense of the meaning literature: An integrative review of meaning making and its effects on adjustment to stressful life events. *Psychological Bulletin,**136*(2), 257–301. 10.1037/a0018301 20192563 10.1037/a0018301

[CR33] Park, C. L. (2013). Religion and meaning. In R. F. Paloutzian & C. L. Park (Eds.), *Handbook of the psychology of religion and spirituality* (2nd ed., pp. 357–379). New York: Guilford Publications.

[CR34] Park, C. L. (2016). Meaning making in the context of disasters. *Journal of Clinical Psychology,**72*(12), 1234–1246. 10.1002/jclp.22270 26900868 10.1002/jclp.22270

[CR35] Park, C. L. (2017). Distinctions to promote an integrated perspective on meaning: Global meaning and meaning-making processes. *Journal of Constructivist Psychology,**30*(1), 14–19. 10.1080/10720537.2015.1119082

[CR36] Park, C. L. (2020). Religiousness and meaning making following stressful life events. In K. E. Vail & C. Routledge (Eds.), *The science of religion, spirituality, and existentialism* (pp. 273–285). Cambridge: Academic Press. 10.1016/B978-0-12-817204-9.00020-2

[CR37] Park, C. L., Currier, J. M., Harris, J. I., & Slattery, J. M. (2017). Trauma, meaning, and spirituality: Translating research into clinical practice. *American Psychological Association*. 10.1037/15961-000

[CR38] Park, C. L., & Van Tongeren, D. R. (2023). Meaning as a framework for integrating positive psychology and the psychology of religiousness and spirituality. In E. B. Davis, E. L. Worthington, & S. A. Schnitker (Eds.), *Handbook of positive psychology, religion, and spirituality* (pp. 83–96). Cham: Springer. 10.1007/978-3-031-10274-5_6

[CR39] Pearce, M. J., Singer, J. L., & Prigerson, H. G. (2006). Religious coping among caregivers of terminally ill cancer patients: Main effects and psychosocial mediators. *Journal of Health Psychology,**11*, 743–759. 10.1177/135910530606662 16908470 10.1177/1359105306066629

[CR40] Podsakoff, P. M., MacKenzie, S. B., Lee, J. Y., & Podsakoff, N. P. (2003). Common method biases in behavioral research: A critical review of the literature and recommended remedies. *Journal of Applied Psychology,**88*(5), 879–903. 10.1037/0021-9010.88.5.879 14516251 10.1037/0021-9010.88.5.879

[CR41] Preacher, K. J., Rucker, D. D., & Hayes, A. F. (2007). Addressing moderated mediation hypotheses: Theory, methods, and prescriptions. *Multivariate Behavioral Research,**42*(1), 185–227. 10.1080/00273170701341316 26821081 10.1080/00273170701341316

[CR42] Sanders, M. P. (2016). *Religious and spiritual struggles among undergraduate students at Wheaton College*. Wheaton College. PhD dissertation.

[CR43] Stauner, N., Exline, J. J., & Wilt, J. A. (2020). Meaning, religious/spiritual struggles, and well-being. In K. E. Vail & C. Routledge (Eds.), *The science of religion, spirituality, and existentialism* (pp. 287–303). Cambridge: Academic Press. 10.1016/B978-0-12-817204-9.00021-4

[CR44] Stearns, M., & McKinney, C. (2021). Parent–child anxiety symptoms in emerging adults: Moderation by gender and religiosity. *Journal of Family Issues,**42*(11), 2691–2710. 10.1177/0192513X20985905

[CR45] Steger, M. F., & Frazier, P. (2005). Meaning in life: One link in the chain from religiousness to well-being. *Journal of Counseling Psychology,**52*(4), 574–582. 10.1037/0022-0167.52.4.574

[CR46] Steger, M. F., Frazier, P., Oishi, S., & Kaler, M. (2006). The meaning in life questionnaire: Assessing the presence of and search for meaning in life. *Journal of Counseling Psychology,**53*(1), 80–93. 10.1037/0022-0167.53.1.80

[CR47] Szcześniak, M., Falewicz, A., Madej, D., Bielecka, G., Pracka, J., & Rybarski, R. (2022). The mediating effect of dispositional gratitude on the relationship between religious struggles and self-esteem: Preliminary results. *Religions,**13*(1), 70. 10.3390/rel13010070

[CR48] Szcześniak, M., & Timoszyk-Tomczak, C. (2020). Religious struggle and life satisfaction among adult Christians: Self-esteem as a mediator. *Journal of Religion and Health,**59*(6), 2833–2856. 10.1007/s10943-020-01082-9 32910280 10.1007/s10943-020-01082-9 PMC7677265

[CR49] Taves, A., Asprem, E., & Ihm, E. (2018). Psychology, meaning making, and the study of worldviews: Beyond religion and non-religion. *Psychology of Religion and Spirituality,**10*(3), 207–217. 10.1037/rel0000201

[CR50] Upenieks, L. (2021). Changes in religious doubt and physical and mental health in emerging adulthood. *Journal for the Scientific Study of Religion,**60*(2), 332–361. 10.1111/jssr.12712

[CR51] Wilt, J. A., Exline, J. J., Grubbs, J. B., Park, C. L., & Pargament, K. I. (2016). God’s role in suffering: Theodicies, divine struggle, and mental health. *Psychology of Religion and Spirituality,**8*(4), 352–362. 10.1037/rel0000058

[CR52] Wilt, J. A., Grubbs, J. B., Pargament, K. I., & Exline, J. J. (2017). Religious and spiritual struggles, past and present: Relations to the big five and well-being. *The International Journal for the Psychology of Religion,**27*(1), 51–64. 10.1080/10508619.2016.1183251

[CR53] You, S., & Lim, S. A. (2019). Religious orientation and subjective well-being: The mediating role of meaning in life. *Journal of Psychology and Theology,**47*(1), 34–47. 10.1177/0091647118795180

[CR54] Zarzycka, B., & Puchalska-Wasyl, M. M. (2020). Can religious and spiritual struggle enhance well-being? Exploring the mediating effects of internal dialogues. *Journal of Religion and Health,**59*(4), 1897–1912. 10.1007/s10943-018-00755-w 30604328 10.1007/s10943-018-00755-w PMC7359172

[CR55] Zarzycka, B., Śliwak, J., Krok, D., & Ciszek, P. (2019). Religious comfort and anxiety in women with cancer: The mediating role of hope and moderating role of religious struggle. *Psycho-Oncology,**28*(9), 1829–1835. 10.1002/pon.5155 31218773 10.1002/pon.5155

[CR56] Zarzycka, B., Tychmanowicz, A., & Krok, D. (2020). Religious struggle and psychological well-being: The mediating role of religious support and meaning making. *Religions,**11*(3), 149. 10.3390/rel11030149

[CR57] Zarzycka, B., & Zietek, P. (2019). Spiritual growth or decline and meaning-making as mediators of anxiety and satisfaction with life during religious struggle. *Journal of Religion and Health,**58*(4), 1072–1086. 10.1007/s10943-018-0598-y 29541972 10.1007/s10943-018-0598-y PMC6606662

